# Neuroprotective Activity of (−)-Epigallocatechin Gallate against Lipopolysaccharide-Mediated Cytotoxicity

**DOI:** 10.1155/2016/4962351

**Published:** 2016-04-12

**Authors:** Jin-Biao Liu, Li Zhou, Yi-Zhong Wang, Xu Wang, Yu Zhou, Wen-Zhe Ho, Jie-Liang Li

**Affiliations:** ^1^Animal Biosafety Level III Laboratory at the Center for Animal Experiment, Wuhan University School of Basic Medical Sciences, Wuhan 430071, China; ^2^Department of Pathology & Laboratory Medicine, Temple University Lewis Katz School of Medicine, Philadelphia, PA 19140, USA; ^3^State Key Laboratory of Virology, Wuhan University, Wuhan 430071, China

## Abstract

Lipopolysaccharide- (LPS-) mediated systemic inflammation plays a critical role in neurodegenerative diseases. The present study was conducted to evaluate the protective effects of epigallocatechin gallate (EGCG), the major component in green tea, on LPS-mediated inflammation and neurotoxicity. LPS treatment of macrophages induced expression of proinflammatory cytokines (TNF-*α*, IL-1*β*, and IL-6). However, EGCG pretreatment of macrophages significantly inhibited LPS-mediated induction of these cytokines. In addition, EGCG significantly diminished LPS-induced inflammatory cytokines in the peripheral mononuclear blood cells (PBMCs). Supernatant from EGCG-pretreated and LPS-activated macrophage cultures was found to be less cytotoxic to neurons than that from non-EGCG-pretreated and LPS-activated macrophage cultures. Furthermore, EGCG treatment of neurons could inhibit LPS-induced production of reactive oxygen species (ROS). Thus EGCG represents a potent and useful neuroprotective agent for inflammation-mediated neurological disorders.

## 1. Introduction

Inflammation plays a critical role in the immunopathogenesis of neurodegenerative diseases such as Parkinson's disease, multiple sclerosis, Alzheimer's disease, and HIV-associated dementia (HAD). Activation of microglia, the intrinsic macrophages in the central nervous system (CNS) [1], is a characteristic feature of neurodegenerative diseases. Mounting evidence clearly indicates that macrophage/microglia activation contributes to inflammation and neuronal injury in the CNS [2, 3]. Lipopolysaccharide (LPS), a major element of Gram-negative bacteria, is a potent activator of immune cells, particularly macrophages and microglia, as it induces expression of proinflammatory cytokines such as TNF-*α*, IL-6, and IL-1*β* [4, 5]. These cytokines have direct or indirect neurotoxic effects on neuronal cells, causing neuronal injury. Microglial activation by LPS plays an important role in the progressive and selective loss of dopaminergic (DA) neurons [6, 7]. Microglia-derived superoxide contributes to about 50% of LPS-induced DA neurotoxicity [8, 9].

Although microglia are vital in the inflammatory process in the CNS, they may have less chance to be activated during a peripheral bacterial infection, as LPS may not be able to enter the CNS due to the blood-brain barrier (BBB). However, monocytes/macrophages in peripheral systems can become activated by LPS, which results in overexpression of proinflammatory cytokines. These cytokines can penetrate BBB and induce an inflammatory environment in the CNS [10]. In addition, activated monocytes in HIV infection have the ability to migrate into the CNS, causing neuronal injury [11]. Furthermore, exposure of macrophages/microglia to invading pathogens leads to the production of ROS, which can benefit the clearance of pathogens but on the other hand cause irreparable damage to neurons [12].

Natural products and dietary components rich in polyphenols have been regarded as promising dietary agents for the prevention and treatment of inflammation-related diseases [13]. (−)-Epigallocatechin gallate (EGCG) is the most abundant catechin in green tea, a beverage widely consumed worldwide. EGCG as a potent antioxidant has been shown to have both anti-inflammatory and antiatherogenic properties in experimental studies conducted* in vitro* and* in vivo* [14, 15]. EGCG was found to inhibit TNF-*α*-induced production of MCP-1/CCL2 from bovine coronary artery endothelial cells, providing direct vascular benefits in inflammatory cardiovascular diseases [16]. It has also been shown that EGCG attenuated the increase in malondialdehyde levels caused by cerebral ischemia and reduced the formation of postischemic brain edema and infarct volume [17]. The neuroprotective effect of EGCG against ischemia-induced brain damage was found, in part, due to the modulation of NOS isoforms and preservation of mitochondrial complex activity and integrity [18]. Thus, the* in vivo* neuroprotective effects of EGCG are not exclusively due to its antioxidant effects but involve more complex signal transduction mechanisms. In addition, the dose of EGCG is vital to be concerned in neuroprotective application, as EGCG presents a biphasic effect based on its concentration-dependent window of pharmacological action. EGCG can act as an antioxidant, reducing ROS at low concentrations [19, 20], and paradoxically may promote the production of ROS and decline of mitochondrial membrane potential and induce apoptosis at high concentrations [21]. In this study, we examined whether EGCG possesses the ability to protect primary human neurons from the macrophages-mediated inflammation and neurotoxicity.

## 2. Materials and Methods

### 2.1. (−)-Epigallocatechin Gallate

EGCG (≥95%) was purchased from Sigma-Aldrich, St. Louis, MO, USA (CAS# 989-51-5). EGCG stock solution was prepared in sterile double distilled water at 20 mM.

### 2.2. Endotoxin-Induced Inflammatory Response and EGCG Treatment

All animal experiments were conducted according to the guidelines for the care and use of laboratory animals and the protocols were approved by the Institutional Animal Care and Use Committee (IACUC) of Animal Biosafety Level III Laboratory at the Center for Animal Experiment. Sixteen adult male Sprague-Dawley rats weighing 200–300 g were obtained from the Center for Animal Experiment, Wuhan University. Briefly, rats were intraperitoneally injected with LPS (from* Escherichia coli*, 055:B5, Invivogen; 1 mg/kg; *n* = 4) or EGCG (5 mg/kg; *n* = 4) or EGCG (5 mg/kg) plus LPS (1 mg/kg; *n* = 4) in 0.1 mL of endotoxin-free phosphate buffered saline (PBS) or 0.1 mL of PBS (*n* = 4). After 24 h, the rats were anesthetized with ketamine and xylazine. Blood samples were collected by cardiac puncture into heparinized syringes. The peripheral blood mononuclear cells (PBMC) were isolated by Ficoll density gradient centrifugation. Following centrifugation (1500 ×g, 30 min, room temperature), PBMC located at the interface were harvested and washed with PBS and lysed with Tri Reagent for RNA extraction.

### 2.3. Monocyte-Derived Macrophage Cultures

Monocytes were obtained from the Path BioResource of the University of Pennsylvania School of Medicine. Blood samples were screened for common blood-borne pathogens and certified to be pathogen-free. Monocytes were isolated by elutriation; the purity of isolated monocytes was higher than 95%. Freshly isolated monocytes were resuspended in DMEM supplemented with 10% fetal bovine serum (FBS), penicillin (100 U/mL), streptomycin (100 *μ*g/mL), and 1% nonessential amino acids. Cells were cultured in 48-well plates (Corning CellBIND Surface, Corning Incorporated, Corning, NY) at 2.5 × 10^5^ cells per well. The medium was half-changed every 48 h. After culture for 7 days, monocytes differentiated into macrophages. Macrophages were incubated with different concentrations of EGCG (0, 10, 20, and 40 *μ*M) for 24 h prior to the treatment with LPS for additional 6 h after which the medium was replenished and cultured for additional 24 h. Supernatant collected from macrophage cultures was used to treat primary human neurons. The cytotoxicity of EGCG to macrophages was measured using a 3-(4, 5-dimethylthiazol-2-yl)-2, 5-diphenyltetrazolium bromide (MTT) assay as previously described [22].

### 2.4. Primary Human Neuron Cultures

Highly enriched neuronal cultures were prepared as described previously [23]. All of the experimental protocols were reviewed and approved by the Institutional Review Board of the University of Minnesota Medical School. Briefly, 11- to 19-week-old fetal brain tissues of aborted fetuses (3 donors) obtained from the Human Embryology Laboratory (University of Washington, Seattle, WA, USA) were dissociated and resuspended in neural basal medium containing B-27 serum-free supplement (contains antioxidants) plus penicillin (100 U/mL) and streptomycin (100 *μ*g/mL). Dispersed cells were plated onto collagen-coated plates (5 × 10^5^ cells/well in 24-well plate) or chamber slides (4 × 10^5^ cells/well in 4-well chambers). On day 12, these brain-cell cultures contained ~70–80% neurons (stained by anti-NeuN or anti-MAP2 antibodies), 15–25% astrocytes (stained by anti-GFAP antibody), and 3–7% microglial cells (stained by anti-CD68 antibody). For highly enriched neuronal cultures, cell cultures were treated with uridine (33.6 *μ*g/mL) and fluorodeoxyuridine (13.6 *μ*g/mL) on day 5, followed by replacement with neural basal medium with B-27 serum-free supplement (contains antioxidants) on day 6 and every 4 days thereafter. Highly purified neuronal cultures contained >95% neurons, 2-3% astrocytes, and 1-2% microglial cells.

### 2.5. Reverse Transcription and Quantitative Real-Time PCR

Total RNA was extracted with Tri Reagent (Sigma-Aldrich) and quantitated by spectrophotometric analysis. Reverse transcription was performed using the AMV transcriptase and RNasin (Promega Co., Madison, WI, USA) according to the manufacturer's instruction. Quantitative real-time PCR (qRT-PCR) was performed with Brilliant SYBR Green Master Mix (Bio-Rad Laboratories, Hercules, CA, USA) described previously [24]. The oligonucleotide primers were synthesized by Integrated DNA Technologies, Inc. (Coralville, IA, USA). The primers that we used for the PCR amplifications are listed as follows: glyceraldehyde 3-phosphate dehydrogenase (GAPDH): 5′-GGTGGTCTCCTCTGA CTTCAACA-3′ (sense) and 5′-GTTGCTGTAGCCAAATTCGTTGT-3′ (antisense); TNF-*α*: 5′-CGAGTGACAAGCCTGTAGC-3′ (sense) and 5′-GGTGTGGGTGAGGAGC ACAT-3′ (antisense); IL-1*β*: 5′-AAGCTGATGGCCCTAAACAG-3′ (sense) and 5′-AGGTGCATCGTGCACATAAG-3′ (antisense); IL-6: 5′-AGGAGACTTGCCTGGTGA AA-3′ (sense) and 5′-CAGGGGTGGTTATTGCATCT-3′ (antisense); iNOS: 5′-GCAGAATGTGACCATCATGG-3′ (sense) and 5′-ACAACCTTGGTGTTGAAGGC-3′ (antisense). All values were calculated using the delta-delta cycle threshold method and expressed as the change relative to the expression of GAPDH.

### 2.6. Immunofluorescence Staining and MAP-2 ELISA

Neuronal cells were seeded on poly-L-lysine coated cover slips in 96-well plates and cultured for two weeks before treatment with LPS or supernatant from LPS-activated macrophage cultures. Cells were then washed with PBS three times and fixed in ice-cold methanol for 5 min. Nonspecific sites were blocked in Block A for 30 min. Cells were then incubated in mouse anti-MAP-2 antibody (1 : 100; Sigma-Aldrich, St. Louis, MO) for 1 h, followed by Alexa 488-conjugated anti-mouse IgG for 30 min. After Hoechst (2 *μ*g/mL) staining, the coverslips were mounted on glass slide and observed under a fluorescence microscope (Olympus IX71). For MAP-2 ELISA, after block, cells were incubated with anti-MAP-2 antibody (1 : 1000) overnight at 4°C. After a wash with PBS, goat *α*-mouse *β*-lactamase TEM-1 (Molecular Probes, Eugene, OR) conjugate (1 : 500; 2 *μ*g/mL) was added into each well and incubated for 30 min and then with fluorocillin green substrate (Invitrogen, Carlsbad, CA) solution in PBS (1 *μ*g/mL) for 1 h. Fluorescence was read at 485/527 nm on a SpectraMax® M3 Multi-Mode Microplate Reader (Molecular Devices, Sunnyvale, CA). The fluorescence of untreated neurons (control) was defined as 100%.

### 2.7. Reactive Oxygen Species (ROS) Detection

Macrophages were pretreated with or without EGCG for 1 h prior to LPS treatment for 24 h. Cells were then washed with serum-free medium and incubated in 10 *μ*M of 2′7′-dichlorofluorescin diacetate (DCFH_2_DA; Molecular Probes) at 37°C for 30 min [24]. After a counterstaining of nuclear with Hoechst 33342 (2 *μ*g/mL) for 5 min and wash, the ROS production was assessed using a fluorescence microscope (Olympus IX71) at 488/527 nm.

### 2.8. Statistical Analysis

Data are expressed as the mean ± SD for at least three independent experiments. Statistical significance was analyzed using Student's *t*-test to compare the means of two groups. For comparison of means of multiple groups, one-way analysis of variance (ANOVA) was performed followed by post-Newman-Keuls test. Differences were considered to be statistically significant when the *P* value was less than 0.05.

## 3. Results

### 3.1. EGCG Attenuates LPS-Induced Inflammatory Cytokines

**Figure 1:**
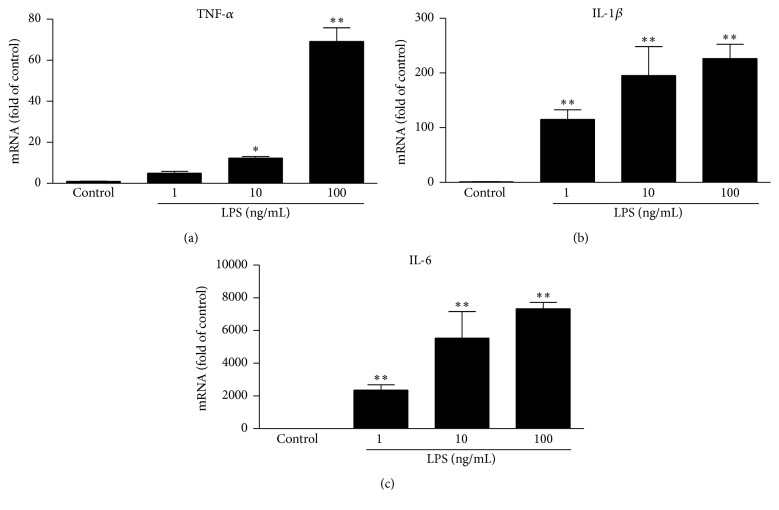
LPS induces the expression of inflammatory cytokines. Human macrophages derived from peripheral blood monocytes were treated with indicated concentrations of LPS for 24 h. Cellular RNA was extracted and subjected to quantitative real-time RT-PCR for TNF-*α* (a), IL-1*β* (b), and IL-6 (c). Data were expressed as mean ± SD of three independent experiments. Data were expressed as mean ± SD of three independent experiments. ^*∗*^ *P* < 0.05; ^*∗∗*^ *P* < 0.01.

**Figure 2:**
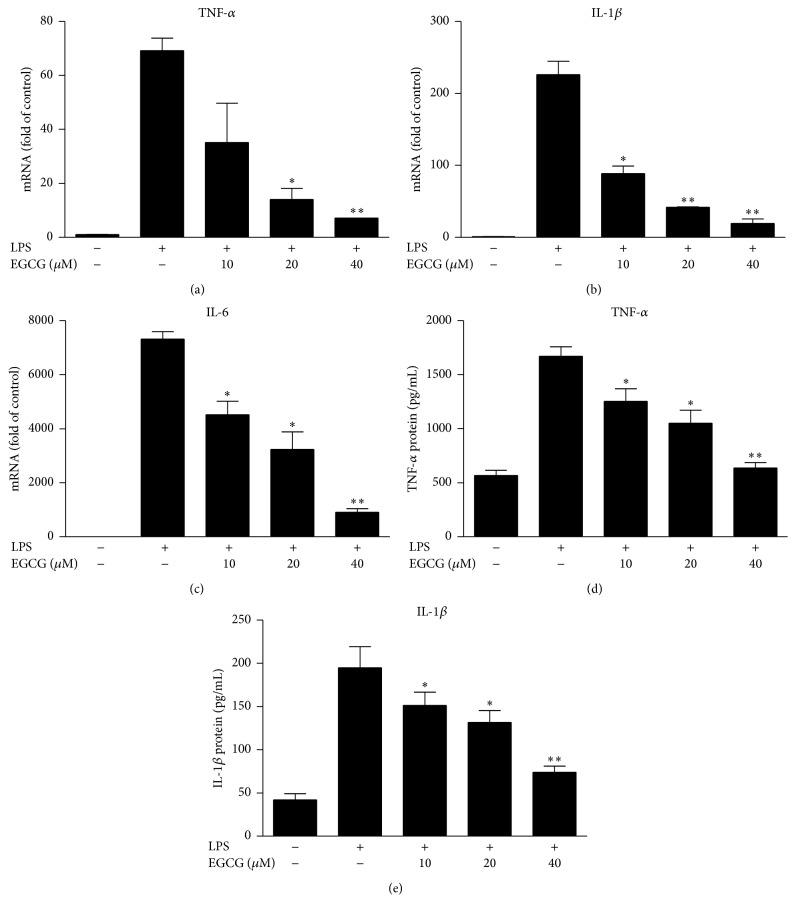
EGCG inhibits LPS-induced expression of inflammatory cytokines. Human macrophages derived from peripheral blood monocytes were treated with indicated concentrations of EGCG for 1 h prior to 100 ng/mL of LPS treatment for additional 24 h. RNA was extracted and subjected to quantitative real-time RT-PCR of TNF-*α*, IL-1*β*, and IL-6. Data were expressed as mean ± SD of three independent experiments. ^*∗*^ *P* < 0.05; ^*∗∗*^ *P* < 0.01, as compared with LPS treated.

**Figure 3:**
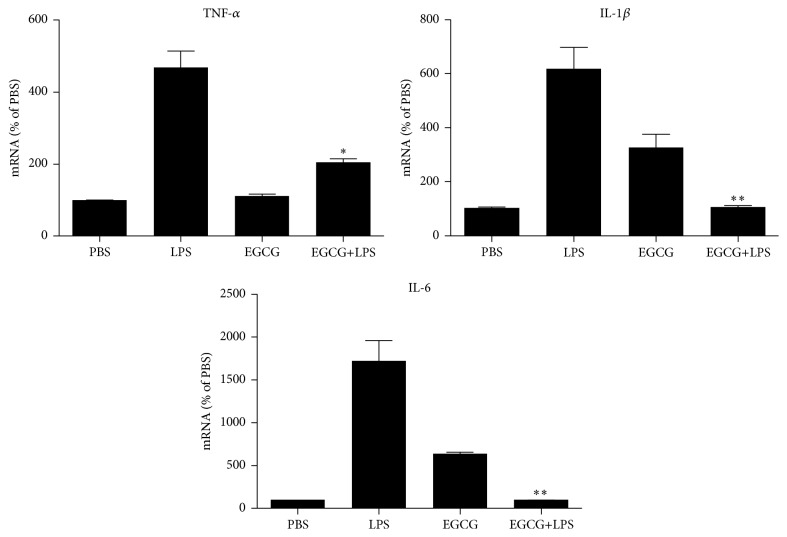
EGCG suppresses LPS-induced expression of inflammatory cytokines in rats. Sprague-Dawley rats were injected with PBS, LPS (1 mg/kg), EGCG (5 mg/kg), or LPS plus EGCG. Twenty-four hours posttreatment, rats (*n* = 4) in each group were sacrificed 24 h after being anesthetized. PBMCs were isolated by Ficoll-plaque and lysed with Tri Reagent. RNA was extracted and subjected to quantitative real-time RT-PCR for TNF-*α*, IL-1*β*, and IL-6. Data were expressed as mean ± SD of 4 animals in each group. ^*∗*^ *P* < 0.05; ^*∗∗*^ *P* < 0.01, as compared with LPS treated.

We first evaluated the* in vitro* effects of EGCG on LPS-induced inflammatory cytokines in primary human macrophages. As shown in Figure 1, LPS treatment of macrophages induced the expression of TNF-*α*, IL-1*β* (600-fold), and IL-6 (1700-fold). However, the expression of these cytokines were significantly reduced in macrophages pretreated with EGCG (Figure 2). This effect of EGCG was dose-dependent (Figure 2) without cytotoxicity (data not shown). We then examined the* in vivo* impact of EGCG on LPS-induced inflammatory cytokines in PBMCs of rats. As shown in Figure 3, LPS challenge of rats induced the expression of TNF-*α* (480-fold), IL-1*β* (600-fold), and IL-6 (1700-fold) in PBMCs. In contrast, EGCG administration significantly attenuated the induction of these cytokines by LPS (Figure 3).

### 3.2. Effect of EGCG on LPS-Induced Neurotoxicity through Macrophages

**Figure 4:**
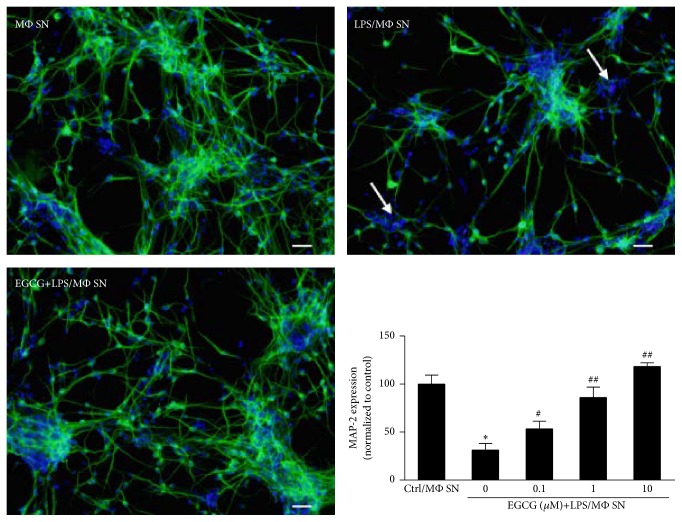
EGCG attenuates the neurocytotoxicity of supernatant from LPS-treated macrophage cultures to human primary neurons. Human macrophages derived from peripheral blood monocytes were treated with 100 ng/mL of LPS for 24 h. For EGCG group, macrophages were pretreated with 10 *μ*M of EGCG for 1 h. Culture supernatant from these macrophage cultures was used to treat human neurons (10%, v/v). The neurocytotoxicity was examined by a cell-based MAP-2 ELISA. Nuclei were counterstained with Hoechst. Data were expressed as mean ± SD and representative data from three independent experiments was shown. Magnification: ×100. ^*∗*^ *P* < 0.05, as compared with control; ^#^ *P* < 0.05; ^##^ *P* < 0.01, as compared with LPS treated. Scale bar = 50 *μ*m.

We next examined the protective effect of EGCG on LPS-induced neurotoxicity.  Figure 4 shows that treatment of primary human neurons with supernatant from LPS-activated macrophage cultures significantly reduced the neuron numbers as identified by MAP-2 immunocytochemistry staining. However, EGCG pretreatment of macrophages remarkably inhibited LPS-induced neurotoxicity (Figure 4).

### 3.3. EGCG Protects Neurons from LPS-Induced Neurotoxicity

**Figure 5:**
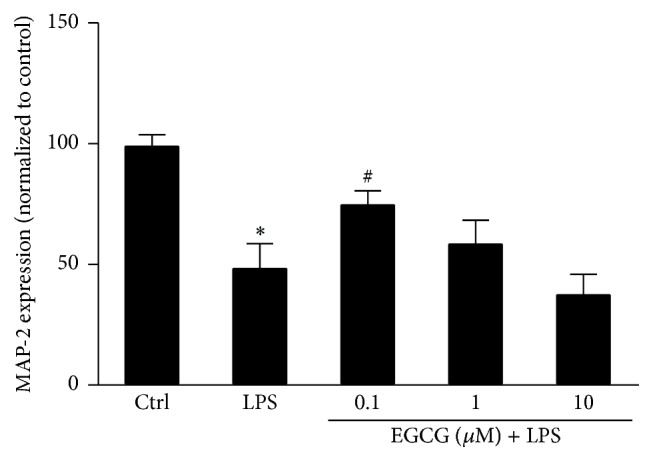
EGCG reduces neurocytotoxicity of LPS to neurons. Primary human neurons were treated with or without 0.1 *μ*M of EGCG for 1 h and then with 100 ng/mL of LPS for additional 48 h. Cells were fixed and stained with anti-MAP-2. Nuclei were counterstained with Hoechst. Data were expressed as mean ± SD of three independent experiments and representative figures were shown. ^*∗*^ *P* < 0.05, as compared with control; ^#^ *P* < 0.05, as compared with LPS only.

From the above we used macrophage cultures to mimic the microglia and we observed that supernatant from LPS-activated macrophages exerted neurotoxicity. Indeed, because it is difficult to obtain pure neuron population (even it can be >95%), there were microglia present in the neuronal cultures albeit at small numbers. We then examined whether LPS direct treatment of the neuronal cultures had neurotoxicity and whether EGCG could protect neurons in this context. When directly added to the neuronal cultures, LPS induced neurotoxicity as evidenced by the reduction of MAP-2 expression (Figure 5). Pretreatment of neurons with EGCG could protect neuronal cells from LPS-mediated cytotoxicity. However, the EGCG concentration (0.1 *μ*M) that can protect neurons directly is much lower than the effective concentrations (10–40 *μ*M) in protecting macrophages from LPS-induced upregulation of cytokines and macrophages-mediated neurocytotoxicity.

### 3.4. EGCG Inhibits LPS-Induced ROS Production in Neurons

**Figure 6:**
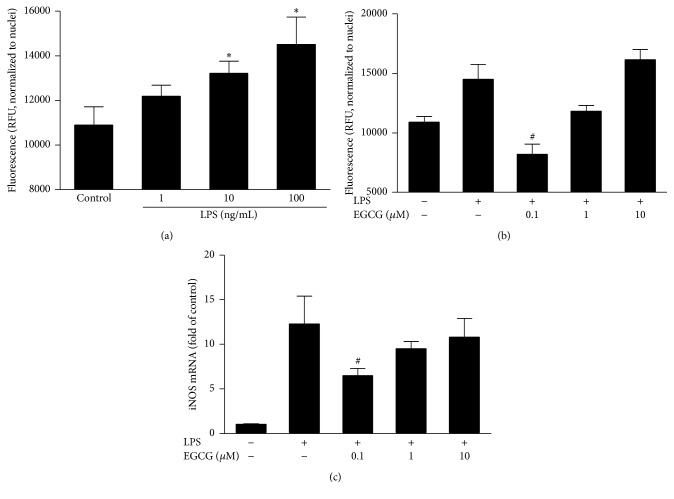
Effect of EGCG on LPS-induced production of ROS in neurons. Primary human neuronal cultures were treated with or without LPS at indicated concentrations for 48 h (a) or with indicated concentrations of EGCG for 1 h prior to 100 ng/mL of LPS treatment for additional 48 h (b). The ROS production was examined by labelling cells with a cell-permeable nonfluorescent probe 2′,7′-dichlorofluorescin diacetate and the fluorescence was measured by a fluorescence microplate reader with excitation at 488 nm and emission at 527 nm. (c) The expression of iNOS in neurons treated with LPS in the presence of indicated concentration of EGCG. Data were expressed as mean ± SD of three independent experiments. ^*∗*^ *P* < 0.05, as compared with control; ^#^ *P* < 0.05, as compared with LPS treated.

To investigate the mechanism(s) of EGCG against LPS-induced direct neurocytotoxicity, we examined the oxidative stress in the neuronal cultures. ROS has been reported as an important mediator for LPS-induced cytotoxicity.  Figure 6(a) shows that LPS treatment of neurons directly induced ROS production and this effect was dose-dependent. EGCG pretreatment inhibited LPS-mediated induction of ROS (Figure 6(b)), as well as the upregulation of iNOS (Figure 6(c)), but the EGCG concentration (0.1 *μ*M) was much lower than that required to exhibit the anti-inflammatory effect in macrophages (10 *μ*M).

## 4. Discussion

It is well known that activated macrophages or microglia produce inflammatory mediators, which has a negative impact on the survival of neurons [1, 25, 26]. Overactivated microglia/macrophages are a chronic source of multiple neurotoxic factors, including TNF-*α*, NO, IL-1*β*, and ROS that can cause progressive neuron damage [26–28]. We found that culture supernatant from LPS-stimulated macrophages exerted neurotoxicity to primary human neurons as evidenced by the reduced expression of specific neuronal marker MAP-2. Systemic inflammatory response which resulted from microbial infection is partly mediated by various pathogen-associated molecular patterns (PAMPs), such as endotoxin [29]. Bacterial endotoxin challenge or exposure plays an important role in inflammation-related damages, including neurodegeneration [2, 30]. Although the production of proinflammatory cytokines (e.g., TNF-*α* and IL-6) by macrophages/microglia is essential in early host defense against infection [31], excessive accumulation of these cytokines disrupts systemic or CNS homeostasis [32–35]. EGCG has been shown to inhibit the induction of TNF-*α* and IL-6 in murine peritoneal macrophages elicited by TLR2/4 signaling [4, 36]. Suppression of IFN-*γ* and IL-6-induced STAT signaling by EGCG has also been reported in mouse splenic monocytes and PBMCs [37, 38]. In addition, our earlier* in vitro* study showed that EGCG pretreatment of human brain microvascular endothelial cells could inhibit LPS-induced expression of inflammatory cytokines [39]. We found that* in vivo* EGCG administration to rat significantly reduced LPS-induced expression of proinflammatory cytokines (TNF-*α*, IL-6, and IL-1*β*) in PBMCs. The underlying mechanism(s) of the EGCG actions has largely been attributed to its suppression of NF-*κ*B activation as well as the negative regulation of cytokine signaling [4, 38–41].

Green tea has been regarded as a nutrient component with possible beneficial effects on neurons although the cellular and molecular mechanism(s) remain unclear. EGCG is the main and most significantly bioactive polyphenol in green tea. We observed that EGCG inhibited the LPS-mediated induction of inflammatory cytokines and attenuated neurotoxicity by LPS-activated macrophages. In addition, EGCG at low dose (0.1 *μ*M) also exerted direct neuroprotective effect against LPS by mitigating the ROS production in neurons. These findings together with studies by others [39, 42, 43] support the notion that EGCG has potential for treating inflammation-induced neuronal injury. Several reports indicated that tea polyphenols can be attained in the brain and exert neuroprotective effect simply by drinking [44–46]. EGCG metabolite could be detected in the brain after oral administration of EGCG to rats [47, 48]. An early observation that there was a wide distribution of labelled EGCG in mouse organs including brain suggests the ability of EGCG to penetrate the BBB [49]. A single, very high oral EGCG dose (500 mg/kg body weight) to rats yielded EGCG concentrations of about 0.5 nmol/g in brain (measured by CL-HPLC) and 20-fold higher in plasma [50]. EGCG was also investigated as a therapeutics adjuvant in the combination therapy to treat multiple sclerosis in mice [51]. However, due to limited systemic absorption, the concentrations of EGCG or EGCG metabolite in the brain are much lower than those in plasma [49].

Interestingly, we revealed that EGCG, at a lower dose of 0.1 *μ*M, but not at higher concentrations (1 and 10 *μ*M), protected neurons from LPS-induced direct neurotoxicity. This neuroprotective activity was concomitantly with the inhibition of ROS production by EGCG in LPS-treated neuronal cultures. Indeed, treatment of neurons with higher concentration (10 *μ*M) of EGCG increased ROS production (data now shown). This biphasic mode of antioxidant and prooxidant activities of EGCG has also been observed in other models [52, 53]. It has been proposed that EGCG exhibits prooxidant and proapoptotic activity at high concentrations, which are responsible for its anticancer cell death effect, while lower doses of EGCG exert neuroprotection against a wide spectrum of neurotoxic compounds [54, 55]. Kucera et al. showed that low doses (<10 *μ*M) of EGCG decreased ROS production whereas EGCG in concentrations of 10 *μ*M and higher induced increase in ROS formation with resultant cellular injury and a decrease in hepatocyte functions. It was revealed that EGCG at high doses led to an uncoupling of mitochondrial oxidative phosphorylation and to damage to the outer mitochondrial membrane [19]. The oxidant activity of EGCG has also been demonstrated in murine macrophages and human leukemic cell lines to increased H_2_O_2_-induced oxidative stress and DNA damage [56, 57]. Catechins, particularly EGCG (100 *μ*M), have been shown to increase the oxidative damage to isolated and cellular DNA after exposure to 8-oxo-7,8-dihydro-2′-deoxyguanosine [58, 59]. The prooxidant activity of EGCG was due to the generation of the hydroxyl radical and hydrogen peroxide in the presence of copper(II) and iron(III), suggesting that antioxidant mechanism of scavenging metals by catechins to stop the formation of free radicals may lead to prooxidant actions on DNA [60]. Excessive EGCG concentrations could also induce toxic levels of ROS* in vivo*. The prooxidative activities and dose-response relationship of EGCG have been implicated in the inhibition of lung cancer cell growth both* in vivo* and* in vitro* [21]. In our* in vivo* experiment, we noticed that EGCG treatment of rat also slightly induced the upregulation of IL-1*β* and IL-6, which might attribute to the prooxidant activity of EGCG. This concentration-dependent biphasic mode is common for some typical radical scavengers and antioxidants, such as ascorbic acid (vitamin C) [61].

In summary, we provide experimental evidence that EGCG attenuates LPS-induced inflammation and LPS-activated macrophage-mediated neurotoxicity at relative higher concentrations (10–40 *μ*M). EGCG at low dose (0.1 *μ*M), but not high concentrations used in macrophages, protects neurons from LPS-induced neurotoxicity and the effect at least partially attributed to the antioxidant activity of EGCG at this concentration. This biphasic mode of action implicates that EGCG may be a good candidate for treatment of inflammation-associated neurodegenerative disorders given the limited availability of EGCG to the brain. Nevertheless, further studies with oral administration of EGCG to suitable animal model are needed.
